# Supplementary material for: Screening and treatment practices for iron deficiency in anaemic pregnant women: A cross-sectional survey of healthcare workers in Nigeria
Source: PLoS One. 2024 Nov 21;19(11):e0310912. doi: 10.1371/journal.pone.0310912 (PMC11581334; doi:10.1371/journal.pone.0310912)
Supplement: S1 File — (DOCX) [file pone.0310912.s001.docx]

**SUPPLEMENTARY MATERIAL 1**

**SURVEY QUESTIONNAIRE**

**TITLE OF RESEARCH:** Current practices on management of iron deficiency anaemia in pregnancy among of maternal healthcare workers in Nigeria

**HREC Approval No.** ............................

**NOTE: Please answer all questions to the best of your ability based on your current practice. Thank you.**

**SECTION A: SOCIODEMOGRAPHIC CHARACTERISTICS/ JOB DESCRIPTION**

1. How old are you now (*at last birthday*)? …........................ years
2. What is your gender?

Male

Female

Prefer not to say

1. In which state do you currently conduct your **primary** work (that is, where do you work most of the time)?

Lagos Kano

1. At what level of care does the health facility where you **primarily** work operate?*

Primary health facility

*(e.g., Primary and Comprehensive Health Centres, Private Clinics)*

Secondary health facility

*(e.g., General Hospitals, Specialist Private Hospitals)*

Tertiary health Institution

*(e.g., Teaching Hospitals)*

Other

*If “other” the next menu opens.*

State the level of care of your facility:________________________________

1. What cadre of maternal health worker are you?*

Doctor

Midwife

Registered nurse

Registered nurse/midwife (double qualified)

1. How long have you been practicing since qualification as a doctor, nurse, or midwife? _______________ years.
2. For how long have you been providing care to pregnant women? _____ years.
3. What type of maternal care do you provide to women? *(Select all that apply)*

Antenatal care

Childbirth care

Postnatal care

Other (specify) __________________________________________

1. On the average, how many pregnant women with **anaemia** do you see per week ?
2. 0 – 5
3. 5 – 10
4. 11 – 15
5. 16 – 20
6. Above 20

** Compulsory fields. Must be completed before you can proceed to the next section.*

**SECTION B: SCREENING AND DIAGNOSIS OF IRON DEFICIENCY ANAEMIA IN PREGNANCY**

**Please answer the following questions about diagnosis of iron deficiency anaemia in pregnancy based on your current practice.**

1. After diagnosis of anaemia in pregnancy with haematocrit (PCV) or haemoglobin concentration, do you screen further to know if it is due to iron deficiency?

Always

Sometimes

Never

*If you select “always” in question 10, skip question 11 and move to the next.*

*If you select “sometimes” in question 10, please answer questions 11 and 12.*

*If you select “never” in question 10, answer question 11 but skip question 12.*

1. What is your reason(s) for **not always** screening pregnant women with anaemia for iron deficiency? (Choose all that apply)

Lack of laboratory facility

Cost

Delay in getting test results

Patient’s refusal

Presumption that anaemia in pregnancy is most often due to iron deficiency

Other (specify) __________________________________________

1. In your current routine practice, which investigation(s) do you use to screen for **iron deficiency** in pregnant women with anaemia? *Select all that apply.*

| **Investigation** | **Yes** |  | **No** |  |  | **Not available** |
| --- | --- | --- | --- | --- | --- | --- |
| a. Complete blood count (CBC) |  |  |  |  |  |  |
| b. Peripheral blood film |  |  |  |  |  |  |
| c. Serum ferritin |  |  |  |  |  |  |
| d. Iron profile |  |  |  |  |  |  |
| e. Rapid diagnostic test |  |  |  |  |  |  |
| f. Other (specify) __________________________________________ | | | | | | |

**SECTION C: PREVENTION AND TREATMENT OF IRON DEFICIENCY ANAEMIA IN PREGNANCY**

**Here, we would like to know how you prevent iron deficiency in pregnancy or how you treat pregnant women with iron deficiency anaemia in your own practice.**

1. What iron preparation(s) do you prescribe for anaemia **prophylaxis (prevention)** in pregnancy? *Select all that apply*.

Oral iron

Intravenous iron

Intramuscular iron

I refer to another health provider for prescription

I do not prescribe iron for prevention

1. What iron preparation(s) do you prescribe for anaemia or iron deficiency anaemia **treatment** in pregnancy? *Select all that apply*.

First line Second line

Oral iron

Intravenous iron

Intramuscular iron

I refer to another health provider for prescription

I do not prescribe iron for treatment

15. **If you select ORAL iron for first-line treatment in question 14 above,** what form of oral iron do you most often use? (*Select all that apply*)

Tablet/ Capsule Liquid

Specify brand(s) of **iron tablet/capsule** used:

Chemiron Fesulf Fergon

Maxiron Bunto Pregnacare Pronatal

Other (specify the brand) ___________________________________________

Specify brand(s) of **liquid iron** used:

Maxiron Chemiron Ranferon Bunto Tothema

Other (specify the brand) ____________________________________________

**If you select INTRAVENOUS iron for first-line treatment in question 14 above,** what form of intravenous iron do you most often use? (Specify brand(s) used):

Iron sucrose Iron dextran (Imferon)

Ferric carboxymaltose Iron isomaltoside

Other (specify the brand) ___________________________________________

**If you select INTRAMUSCULAR iron for first-line treatment in question 14 above,** what form of intramuscular iron do you most often use? (Specify brand(s) used):

Iron sucrose Iron dextran (Imferon)

Ferric carboxymaltose Iron isomaltoside

Other (specify the brand) ___________________________________________

*Please answer Question 16 only* ***if you have selected oral iron*** *either as first or second-line treatment in Question 14 above.*

1. At what dose do you most commonly give oral iron preparations such as ferrous sulphate 200mg, ferrous gluconate 300mg, or ferrous fumarate 210mg to pregnant women for treatment of iron deficiency anaemia? (*Select one*)

One tablet daily

One tablet twice daily

One tablet 3 times daily

2 tablets 3 times daily

Other (specify) _________________________________________

1. What influences your decision to **treat** for iron deficiency anaemia? *Select all that apply.*

- Laboratory test result suggesting low iron levels
- I treat selected cases empirically based on suspicion
- I treat all pregnant women who are anaemic as cases of iron deficiency
- Woman’s willingness to pay for additional tests
- Other (specify) _______________________________________

1. At what trimester of pregnancy would you prescribe the following drugs for treatment of iron deficiency anaemia? (*Select all that apply for each drug that you prescribe*)

**Oral iron** – First trimester Second trimester Third trimester

**Intramuscular iron** – First trimester Second trimester Third trimester

**Intravenous iron** – First trimester Second trimester Third trimester

1. If you use intravenous iron for iron deficiency anaemia treatment in pregnancy, based on your practice and experience, what do you consider to be the **advantage(s)** of using **intravenous iron** compared to oral iron? *Select all that apply.*

- Faster improvement in anaemia treatment compared to oral iron
- Beneficial when oral iron fails
- Easy to administer
- Fast to administer
- Faster correction of iron deficiency anaemia in late pregnancy
- Preferable to most women
- Greater tolerance compared to oral iron
- Other (specify) ____________________________________________

1. If you use intravenous iron for iron deficiency anaemia treatment in pregnancy, based on your practice and experience, what do you consider to be the **disadvantage(s)** of using **intravenous iron** compared to oral iron? *Select all that apply.*

- Need for venepuncture
- Time consuming in terms of administration
- Requires manpower
- Expensive
- Safety in pregnancy in doubt
- Women refuse intravenous iron
- Worse side effect profile compared to oral iron
- Other (specify) _________________________________________

**SECTION D: ANAEMIA TREATMENT AND INFECTION**

**Now we** **would like to ask you a few final questions about anaemia treatment when there is an ongoing infection.**

1. Do you normally **treat** pregnant **anaemic women with iron supplements** when they have an **ongoing infection** such as malaria or sepsis?

Yes

No

*If you select “NO” in question 21 above, skip questions 22 and 23 and answer question 24.*

*If you select “YES” in question 21 above, answer questions 22 and 23, but skip question 24 only.*

1. **If YES to question 21**, explain why you will give iron preparation to a pregnant woman who has anaemia and infection at the same time

_______________________________________________________________

1. With what **do you treat** anaemia in pregnancy, in the presence of an ongoing infection? (*Select all that apply*)

Oral iron

Intramuscular iron

Intravenous iron

Blood transfusion

Other _________________________

1. **If NO to question 21**, why do you prefer **NOT to treat** anaemia in pregnancy with iron preparation when there is an infection at the same time? State your reason.

______________________________________________________________

***Thank you for participating in this survey!***

*The results will be available by December 2022 and can be shared via email. If you would like to receive the results, please send an email to Dr Ochuwa Babah at* [*ochuwab@yahoo.co.uk*](mailto:ochuwab@yahoo.co.uk) *with the subject line “Online survey results on iron deficiency anaemia in pregnancy – interested.*
