# Supplementary material for: Screening and treatment practices for iron deficiency in anaemic pregnant women: A cross-sectional survey of healthcare workers in Nigeria
Source: PLoS One. 2024 Nov 21;19(11):e0310912. doi: 10.1371/journal.pone.0310912 (PMC11581334; doi:10.1371/journal.pone.0310912)
Supplement: S2 File — (DOCX) [file pone.0310912.s002.docx]

**SUPPLEMENTARY MATERIAL 2**

**INFORMATION SHEET**

**TITLE OF RESEARCH:** Current practices on management of iron deficiency anaemia in pregnancy among of maternal healthcare workers in Nigeria

**LUTH HREC Approval No.** ADM/DSCST/HREC/APP/4864

**NAME & AFFILIATION OF RESEARCHER:** The study which is part of my PhD research will be coordinated by me, Dr Ochuwa Babah, Dept. of Obstetrics and Gynaecology, College of Medicine of University of Lagos and Lagos University Teaching Hospital, Idi-Araba, Lagos, Nigeria.

**PURPOSE OF THE RESEARCH:** Anaemia in pregnancy is a public health burden and can affect women and/or their babies. It is a leading cause of maternal death in Nigeria. There are different causes of anaemia. In Nigeria there is paucity of data related to practices on anaemia treatment, hence this study. We believe the findings of this study can help inform protocol development for treatment of iron deficiency anaemia in pregnancy in Nigeria. The study will assess how you make the diagnosis in your own practice, and how you treat patients with iron deficiency anaemia.

**PROCEDURE OF THE RESEARCH:** The research will only involve you filling out a survey form online after consenting, by clicking I agree to participate. It will take about 5 – 10 minutes of your time.

**POTENTIAL BENEFITS:** The study will enable us to have objective information about current practices related to the diagnosis and treatment of iron deficiency anaemia in pregnancy in Nigeria. The findings might also influence protocol development on treatment of iron deficiency anaemia in pregnancy in future.

**POTENTIAL RISKS**: The research poses negligible risk as it is questionnaire-based and does not involve any form of intervention or invasive procedure.

**CONFIDENTIALITY:** There will be no identifier in the survey form so your answers can never be linked to you in any way. All information obtained will be kept strictly confidential.

**WILLINGNESS TO PARTICIPATE:** Your participation in this research is entirely voluntary and if you choose not to participate, no punishment will be attached to your decision. You will not be paid any fees for participating in this research. You can choose to withdraw from the research at any time.

**WHAT HAPPENS TO RESEARCH PARTICIPANTS AND COMMUNITY WHEN THE RESEARCH IS OVER:** The researcher will inform you of the outcome of the research if you wish to be notified. To get information on the research outcome, feel free to send a separate message to the researcher’s email address which appears at the end of the consent section. There is no conflict of interest whatsoever in conducting this study.

**CONSENT FORM**

**TITLE OF RESEARCH:** Current practices on management of iron deficiency anaemia in pregnancy among of maternal healthcare workers in Nigeria

**LUTH HREC Approval No.** ADM/DSCST/HREC/APP/4864

**SCREENING FOR ELIGIBILITY**

Are you a medical doctor, nurse or midwife based in Nigeria who provides antenatal care to pregnant women? This means that you have provided clinical care to pregnant women yourself or supervised the provision of care by others in the past 12 months.

Yes No

(*For the online version if “Yes” is selected for the question above, the consent section opens, and if “No” is selected the survey automatically closes with the message “Sorry you are not eligible to participate in this survey. Thank you”)*

**CONSENT**

Dear Sir/ Madam,

I am a Lecturer at the Department of Obstetrics and Gynaecology, College of Medicine, University of Lagos, Idi-Araba, Lagos, Nigeria. I am conducting this research to assess maternal healthcare worker awareness and practices adopted for the management of iron deficiency anaemia in pregnant Nigerian women, as part of my doctoral study on iron deficiency anaemia in pregnancy. This study will potentially help inform policy and practice as it relates to management of iron deficiency anaemia in pregnancy. It will take only about 10 - 15 minutes of your time to complete this online survey. Your decision to participate in this research is voluntary. Anonymity and confidentiality of data collected will be maintained.

If you agree to take part in this research, kindly click ‘’Yes’’ below to consent and proceed or click ‘’No’’ to decline.

Thank you.

Yes No

(*For the online version if “Yes” is selected for the question above, the survey opens, and if “No” is selected the survey automatically closes with the message “You are permitted to end the survey here. Thank you”)*
