# Supplementary material for: Screening and treatment practices for iron deficiency in anaemic pregnant women: A cross-sectional survey of healthcare workers in Nigeria
Source: PLoS One. 2024 Nov 21;19(11):e0310912. doi: 10.1371/journal.pone.0310912 (PMC11581334; doi:10.1371/journal.pone.0310912)
Supplement: S1 Table — (DOCX) [file pone.0310912.s003.docx]

**SUPPLEMENTARY MATERIAL 3**

**Table SM1. Type and dosages of oral iron prescribed by maternal healthcare workers**

| **Variable** | **Total responses** | **% Total (95%CI)**  **n = 370** | **% Doctors (95%CI)**  **n = 132** | **% Registered nurses and/or midwives (95%CI)**  **n = 238** | **p-value** |
| --- | --- | --- | --- | --- | --- |
| **Type of oral iron prescribed** |  |  |  |  |  |
| Tablet/capsule | 365 | 69.8  (64.9-74.5) | 67.1  (58.4-75.0) | 71.8  (65.6-77.4) | 0.407^ |
| Liquid | 365 | 40.0  (35.0-45.2) | 42.4  (33.9-51.3) | 38.3  (32.1-44.8) | 0.330^ |
| **Dosage of oral iron prescribed*** |  |  |  |  |  |
| One tablet daily | 288 | 16.8  (12.6-21.6) | 12.7  (7.0-20.7) | 19.9  (14.4-26.4) |  |
| One tablet twice daily | 288 | 32.4  (27.1-38.2) | 27.6  (19.3-37.2) | 36.2  (29.3-43.6) |  |
| One tablet 3 times daily | 288 | 42.6  (36.9-48.6) | 52.7  (42.6-62.5) | 35.4  (28.5-42.8) | 0.285^ |
| Two tablets 2 times daily | 288 | 0.6  (0.1-2.4) | 0.7  (0.0-4.8) | 0.0 |  |
| Two tablets 3 times daily | 288 | 7.5  (4.7-11.2) | 6.3  (2.4-12.8) | 8.5  (4.9-13.5) |  |

**Total comprises all doctors, nurses, and midwives only. The dosage of oral iron stated are for iron tablets like ferrous sulphate 200mg containing 65mg elemental iron, ferrous gluconate 300mg containing 35mg, or ferrous fumarate 210mg containing 68mg elemental iron prescribed to pregnant women for the treatment of iron deficiency anaemia by maternal healthcare workers. Pearson chi-square test used was for hypothesis testing. p-value compares the prescription practices of doctors versus nurses/midwives.*
