# Supplementary material for: Screening and treatment practices for iron deficiency in anaemic pregnant women: A cross-sectional survey of healthcare workers in Nigeria
Source: PLoS One. 2024 Nov 21;19(11):e0310912. doi: 10.1371/journal.pone.0310912 (PMC11581334; doi:10.1371/journal.pone.0310912)
Supplement: S2 Table — (DOCX) [file pone.0310912.s004.docx]

**SUPPLEMENTARY MATERIAL 4**

**Table SM2. Dosage of iron tablets commonly prescribed for the treatment of iron deficiency anaemia**

| **Brand of oral iron** | **Percentage of total** |
| --- | --- |
| ***Ferrous sulphate 200mg (n=156)*** |  |
| One tablet daily | 9.5 (5.4-15.2) |
| One tablet twice daily | 33.1 (25.8-41.1) |
| One tablet 3 times daily* | 48.5 (40.5-56.7) |
| Two tablets 2 times daily* | 0.0 |
| Two tablets 3 times daily* | 8.8 (4.9-14.4) |
| ***Ferrous gluconate 210mg (n=102)*** |  |
| One tablet daily | 9.8 (4.8-17.3) |
| One tablet twice daily | 38.8 (29.3-48.9) |
| One tablet 3 times daily | 47.4 (37.4-57.5) |
| Two tablets 2 times daily* | 1.1 (0.0-5.5) |
| Two tablets 3 times daily* | 2.9 (0.6-8.3) |

*Ferrous sulphate 200mg contains 65mg elemental iron and ferrous gluconate 300mg contains 35mg. Percentages are weight-adjusted values.*

**Indicates dosages above World Health Organization recommended 120mg elemental iron daily for treatment of iron deficiency anaemia during pregnancy.*
