# Supplementary material for: Screening and treatment practices for iron deficiency in anaemic pregnant women: A cross-sectional survey of healthcare workers in Nigeria
Source: PLoS One. 2024 Nov 21;19(11):e0310912. doi: 10.1371/journal.pone.0310912 (PMC11581334; doi:10.1371/journal.pone.0310912)
Supplement: S3 Table — (DOCX) [file pone.0310912.s005.docx]

**SUPPLEMENTARY MATERIAL 6**

**Table SM3. Perception of maternal health workers regarding the use of intravenous iron for treatment in pregnancy (n=467)**

| **Perceived advantages*** | **Percentage (95%CI)** |
| --- | --- |
| Faster improvement in anaemia treatment compared to oral iron | 47.3 (42.7-52.0) |
| Suitable when there is a need for faster correction of iron deficiency anaemia in late pregnancy | 34.5 (30.1-39.0) |
| Beneficial as second-line treatment when oral iron fails | 31.2 (27.0-35.6) |
| Greater tolerance compared to oral iron | 12.3 (9.4-15.6) |
| Easy for the health worker to administer | 5.6 (3.7-8.1) |
| Fast for the health worker to administer | 5.5 (3.6-8.0) |
| Preferable to most women | 2.4 (1.2-4.3) |
| **Perceived disadvantages**** | **Percentage (95%CI)** |
| Expensive | 40.1 (35.6-44.7) |
| Need for venipuncture | 39.8 (35.3-44.4) |
| Requires human resources | 25.9 (22.0-30.2) |
| Time-consuming in terms of administration | 19.7 (16.2-23.6) |
| Worse side effect profile compared to oral iron | 19.1 (15.6-23.0) |
| Women refuse intravenous iron | 13.1 ( 10.1-16.5) |
| Safety in pregnancy in doubt | 6.3 (4.3-8.9) |

*Figures are percentages of weighted sample (95% confidence intervals). Of the unweighted sample of the 467 maternal healthcare workers: *For perceived advantages, 113 (24.2%) gave three or more reasons, 49 (10.5%) two reasons, 104 (22.3%) gave only one reason, and 201 (43.0) did not give any reason. **For perceived disadvantages, 135 (28.9%) gave three or more reasons, 45 (9.6%) two reasons, 85 (18.2%) gave only one reason, and 202 (43.3) did not give any reason.*
