# Supplementary material for: Screening and treatment practices for iron deficiency in anaemic pregnant women: A cross-sectional survey of healthcare workers in Nigeria
Source: PLoS One. 2024 Nov 21;19(11):e0310912. doi: 10.1371/journal.pone.0310912 (PMC11581334; doi:10.1371/journal.pone.0310912)
Supplement: S4 Table — (DOCX) [file pone.0310912.s006.docx]

**SUPPLEMENTARY MATERIAL 7**

**Table SM4: Maternal health workers’ approach towards treating iron deficiency anaemia when there is concomitant infection**

| **Practice when there is concomitant infection** | **Total number** | **% Total** | **% Doctors** | **% Registered nurses and/or midwives** | **% CHEWs** | **p-value^a^** |
| --- | --- | --- | --- | --- | --- | --- |
| Proportion who prescribes iron during concomitant infection | 379 | 52.8  (47.6-57.9) | 43.9  (34.4-53.6) | 61.7  (54.5-68.6) | 35.9  (25.1-47.8) | 0.074^ |
| Method of treatment during infection |  |  |  |  |  |  |
| Oral iron | 199 | 42.1  (46.1-58.0) | 53.4  (41.0-65.5) | 51.0  (42.7-59.3) | 56.0  (43.4-68.0) | 0.438^ |
| Intravenous iron | 199 | 3.5  (1.7-6.3) | 1.8  (0.1-8.4) | 4.3  (1.7-9.0) | 4.4  (0.9-12.3) | 1.000^#^ |
| Intramuscular iron | 199 | 11.4  (8.0-15.7) | 6.4  (1.9-15.0) | 14.9  (9.6-21.6) | 4.4  (0.9-12.3) | **0.044**^ |
| Blood transfusion | 199 | 38.4  (32.7-44.3) | 40.1  (28.5-52.6) | 39.4  (31.5-47.8) | 12.0  (5.4-22.2) | 0.531^ |
| Dietary modification | 199 | 0.4  (0.0-2.1) | 0.0 | 0.6  (0.0-3.6) | 1.1  (0.0-7.3) | 1.000^#^ |

*^a^p-value compares prescription practices by doctors versus nurses/midwives only for pregnant women with anaemia and concomitant infection. Hypothesis testing was done using ^Pearson chi-square test and ^#^Fisher’s exact test.*
