# Supplementary material for: Screening and treatment practices for iron deficiency in anaemic pregnant women: A cross-sectional survey of healthcare workers in Nigeria
Source: PLoS One. 2024 Nov 21;19(11):e0310912. doi: 10.1371/journal.pone.0310912 (PMC11581334; doi:10.1371/journal.pone.0310912)
Supplement: S1 Fig — (DOCX) [file pone.0310912.s007.docx]

**SUPPLEMENTARY MATERIAL 5**


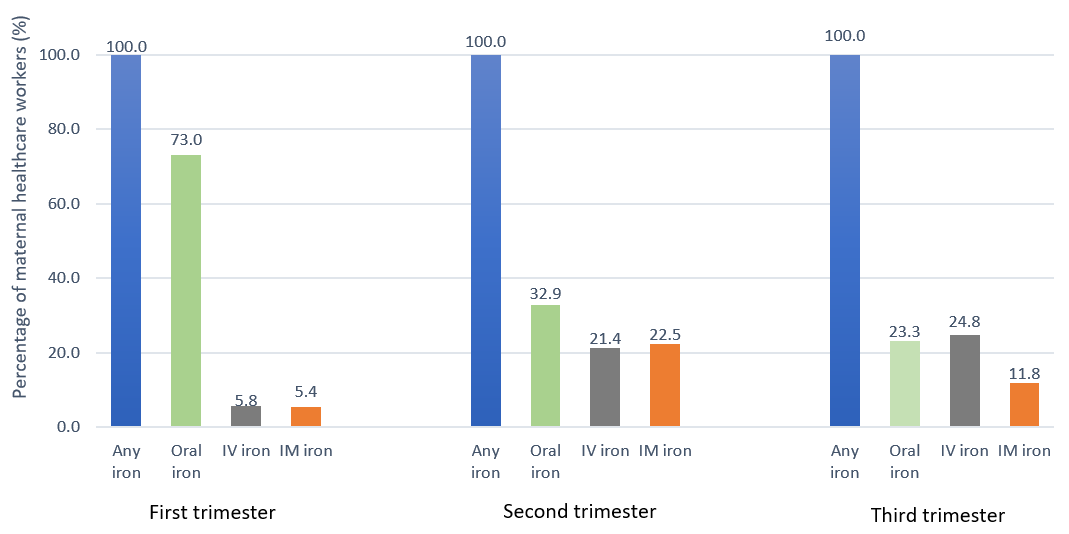


*Percentages are of weighted sample, n = 449. IV – intravenous, IM – intramuscular. Any iron can be oral or intravenous or intramuscular iron.*

**Figure SM1. Maternal healthcare workers' willingness to prescribe various formulations of iron in each trimester of pregnancy**
