# Supplementary material for: Screening and treatment practices for iron deficiency in anaemic pregnant women: A cross-sectional survey of healthcare workers in Nigeria
Source: PLoS One. 2024 Nov 21;19(11):e0310912. doi: 10.1371/journal.pone.0310912 (PMC11581334; doi:10.1371/journal.pone.0310912)
Supplement: S2 Fig — (DOCX) [file pone.0310912.s008.docx]

**SUPPLEMENTARY MATERIAL 8**

***
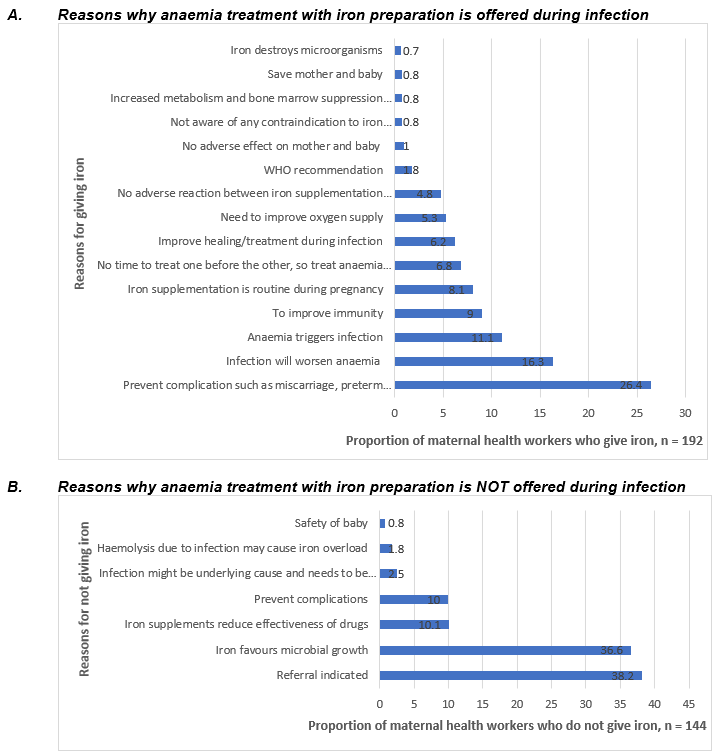
***

*Proportion of sub-group of maternal health workers who would give iron to anaemic pregnant women when there is an ongoing infection (A) and sub-group of maternal health workers who would not give iron supplements when there is an ongoing infection (B), are presented as percentage of weighted samples in each sub-group.*

**Figure SM2: Maternal health workers’ perception towards treating iron deficiency anaemia when there is concomitant infection**
