# Supplementary material for: Screening and treatment practices for iron deficiency in anaemic pregnant women: A cross-sectional survey of healthcare workers in Nigeria
Source: PLoS One. 2024 Nov 21;19(11):e0310912. doi: 10.1371/journal.pone.0310912 (PMC11581334; doi:10.1371/journal.pone.0310912)
Supplement: Attachment — Submitted filename: RESPONSE TO REVIEWERS - 17-06-2024.docx [file pone.0310912.s009.docx]

**Manuscript ID:** PONE-D-24-09943

**Title:** Screening and treatment practices for iron deficiency anaemia during pregnancy: a cross-sectional survey of healthcare workers in Nigeria

Dear Editor,

Thank you for a quick response. Thank you to the reviewers also for their time and critical review of this manuscript. The comments were all noted and have been addressed with point-by-point response to each comment below (in blue font).

***Please note that all page and line numbers listed in our responses are of the revised manuscript with track changes.***

Thank you.

Best regards,

Ochuwa Babah

**From the editor:**

Dear Dr. Babah,

Thank you for submitting your manuscript to PLOS ONE. After careful consideration, we feel that it has merit but does not fully meet PLOS ONE’s publication criteria as it currently stands. Therefore, we invite you to submit a revised version of the manuscript that addresses the points raised during the review process.

Please see the comments of two reviewers below. In addition, please include the full ethics statement in the Methods section, and please ensure that you have reported all covariates and confounding variables.

**Response:**

1. Ethics statements have been moved from the section on declaration to the section on ethical consideration in the methodology (Page 13, lines 287-299).

2. Manuscript has also been checked to ensure all covariates and confounding variables are reported.


**Please include the following items when submitting your revised manuscript:**

- **A rebuttal letter that responds to each point raised by the academic editor and reviewer(s). You should upload this letter as a separate file labeled 'Response to Reviewers'.**
- **A marked-up copy of your manuscript that highlights changes made to the original version. You should upload this as a separate file labeled 'Revised Manuscript with Track Changes'.**

**Response**: A response letter to the reviewers’ comments has been uploaded to portal and a marked copy of the manuscript (tracked) has been uploaded to portal.

- An unmarked version of your revised paper without tracked changes. You should upload this as a separate file labeled 'Manuscript'.

**Response**

A clean copy of the manuscript has been uploaded to portal.

If you would like to make changes to your financial disclosure, please include your updated statement in your cover letter. Guidelines for resubmitting your figure files are available below the reviewer comments at the end of this letter.

**Response**

We do not have plans to make any change to the financial disclosure.

If applicable, we recommend that you deposit your laboratory protocols in protocols.io to enhance the reproducibility of your results. Protocols.io assigns your protocol its own identifier (DOI) so that it can be cited independently in the future. For instructions see: <https://journals.plos.org/plosone/s/submission-guidelines#loc-laboratory-protocols>. Additionally, PLOS ONE offers an option for publishing peer-reviewed Lab Protocol articles, which describe protocols hosted on protocols.io. Read more information on sharing protocols at <https://plos.org/protocols?utm_medium=editorial-email&utm_source=authorletters&utm_campaign=protocols>.

**Response**

Thank you for the suggestion. We did not find a need to publish the protocol for this study since it is not laboratory-based research. However, to enhance reproducibility of the research, we have extensively described how the research was conducted in the Methods section, and also include the complete survey questionnaire and informed consent form in the appendix.

**Journal requirements:**

1. Please ensure that your manuscript meets PLOS ONE's style requirements, including those for file naming. The PLOS ONE style templates can be found at

<https://journals.plos.org/plosone/s/file?id=wjVg/PLOSOne_formatting_sample_main_body.pdf> and

<https://journals.plos.org/plosone/s/file?id=ba62/PLOSOne_formatting_sample_title_authors_affiliations.pdf>

**Response**

Thank you. The journal style is well noted. We have gone through the entire manuscript to ensure that the style is in order. Revisions have been made where necessary. These include recommended symbol labels for authorship (Page 1, lines 4 and 5) and use of email only for corresponding author (Page 1, lines 15-20). In addition, we have ensured that all titles and sub-titles have the recommended fonts, and that tables, figures and supplementary information files, tables and figures are presented and labelled according to the journal requirements.

2. In this instance it seems there may be acceptable restrictions in place that prevent the public sharing of your minimal data. However, in line with our goal of ensuring long-term data availability to all interested researchers, PLOS’ Data Policy states that authors cannot be the sole named individuals responsible for ensuring data access (http://journals.plos.org/plosone/s/data-availability#loc-acceptable-data-sharing-methods).

Data requests to a non-author institutional point of contact, such as a data access or ethics committee, helps guarantee long term stability and availability of data. Providing interested researchers with a durable point of contact ensures data will be accessible even if an author changes email addresses, institutions, or becomes unavailable to answer requests.

Before we proceed with your manuscript, please also provide non-author contact information (phone/email/hyperlink) for a data access committee, ethics committee, or other institutional body to which data requests may be sent. If no institutional body is available to respond to requests for your minimal data, please consider if there any institutional representatives who did not collaborate in the study, and are not listed as authors on the manuscript, who would be able to hold the data and respond to external requests for data access? If so, please provide their contact information (i.e., email address). Please also provide details on how you will ensure persistent or long-term data storage and availability.

**Response**

Thank you. We have deposited the dataset for this study in Open Science Framework. The data is accessible at: <https://osf.io/ybcdq/>

This has also been stated in the manuscript (Page 25, paragraph 563-565), with the link to the dataset.

3. Your ethics statement should only appear in the Methods section of your manuscript. If your ethics statement is written in any section besides the Methods, please move it to the Methods section and delete it from any other section. Please ensure that your ethics statement is included in your manuscript, as the ethics statement entered into the online submission form will not be published alongside your manuscript.

**Response**

Thank you. The ethics statement has been removed from the Declarations section and transferred to the Methods section (Page 13, lines 287-299).

4. We note that Figure 1 in your submission contain [map/satellite] images which may be copyrighted. All PLOS content is published under the Creative Commons Attribution License (CC BY 4.0), which means that the manuscript, images, and Supporting Information files will be freely available online, and any third party is permitted to access, download, copy, distribute, and use these materials in any way, even commercially, with proper attribution. For these reasons, we cannot publish previously copyrighted maps or satellite images created using proprietary data, such as Google software (Google Maps, Street View, and Earth). For more information, see our copyright guidelines: http://journals.plos.org/plosone/s/licenses-and-copyright.

We require you to either (1) present written permission from the copyright holder to publish these figures specifically under the CC BY 4.0 license, or (2) remove the figures from your submission:

a. You may seek permission from the original copyright holder of Figure 1 to publish the content specifically under the CC BY 4.0 license.

We recommend that you contact the original copyright holder with the Content Permission Form (http://journals.plos.org/plosone/s/file?id=7c09/content-permission-form.pdf) and the following text:

“I request permission for the open-access journal PLOS ONE to publish XXX under the Creative Commons Attribution License (CCAL) CC BY 4.0 (http://creativecommons.org/licenses/by/4.0/). Please be aware that this license allows unrestricted use and distribution, even commercially, by third parties. Please reply and provide explicit written permission to publish XXX under a CC BY license and complete the attached form.”

Please upload the completed Content Permission Form or other proof of granted permissions as an "Other" file with your submission.

In the figure caption of the copyrighted figure, please include the following text: “Reprinted from [ref] under a CC BY license, with permission from [name of publisher], original copyright [original copyright year].”

b. If you are unable to obtain permission from the original copyright holder to publish these figures under the CC BY 4.0 license or if the copyright holder’s requirements are incompatible with the CC BY 4.0 license, please either i) remove the figure or ii) supply a replacement figure that complies with the CC BY 4.0 license. Please check copyright information on all replacement figures and update the figure caption with source information. If applicable, please specify in the figure caption text when a figure is similar but not identical to the original image and is therefore for illustrative purposes only.

The following resources for replacing copyrighted map figures may be helpful:

USGS National Map Viewer (public domain): http://viewer.nationalmap.gov/viewer/

The Gateway to Astronaut Photography of Earth (public domain): http://eol.jsc.nasa.gov/sseop/clickmap/

Maps at the CIA (public domain): https://www.cia.gov/library/publications/the-world-factbook/index.html and https://www.cia.gov/library/publications/cia-maps-publications/index.html

NASA Earth Observatory (public domain): http://earthobservatory.nasa.gov/

Landsat: http://landsat.visibleearth.nasa.gov/

USGS EROS (Earth Resources Observatory and Science (EROS) Center) (public domain): http://eros.usgs.gov/#

Natural Earth (public domain): <http://www.naturalearthdata.com/>

**Response**

Thank you for bringing forth this observation on the need to cite the source of the map in Figure 1. The previous image was created using an editable map obtained from a colleague. Unfortunately, we could not trace the source. So, we have now created a new map of Nigeria, showing the two states where the study was conducted, on Microsoft Excel. The facility for generating map on Excel spreadsheet is powered by Bing © Microsoft OpenStreetMap and has been cited in Figure 1, with the date the map was created indicated (*Figure 1*).

5. Please review your reference list to ensure that it is complete and correct. If you have cited papers that have been retracted, please include the rationale for doing so in the manuscript text, or remove these references and replace them with relevant current references. Any changes to the reference list should be mentioned in the rebuttal letter that accompanies your revised manuscript. If you need to cite a retracted article, indicate the article’s retracted status in the References list and also include a citation and full reference for the retraction notice.

[Note: HTML markup is below. Please do not edit.]

**Response**

Thank you. This is noted. In addition to the newly added reference the map (above), we have added one new reference (Reference 28) in-text in the methodology and to the reference list while expanding the section on sample size (Page 6, lines 128-130, and Page 28, lines 653-655 respectively). We have also cross-checked to ensure all references are correctly cited.

**Reviewers' comments**

**Reviewer #1**:

Is it "Screening and treatment practices for IDA during pregnancy among ANC visitors or among anaemic pregnant mothers?" Clearly state and write it from the result and discussion sections depending on your aim.

**Response**

Thank you for this observation. The title has been revised to enhance clarity and in accordance with the study objectives, the results and discussion. We have changed it from “*Screening and treatment practices for iron-deficiency anaemia during pregnancy: a cross-sectional survey of healthcare workers in Nigeria*” to “**Screening and treatment practices for iron-deficiency in anaemic pregnant women: a cross-sectional survey of healthcare workers in Nigeria**” (Page 1, lines 1-3).This has also been aligned with the objectives for enhance clarity (Page 2, line 31 under Abstract, and Page 5, line 99 under introduction).

You state various keywords even more than the recommended.

**Response**

Thank you for drawing our attention to this. We were unsure of the maximum number of keywords allowed by the journal because we could not access the information, even now. However, we have now reduced the number of keywords from thirteen to eight (Page 3, lines 55-57). The keywords are listed in order of importance, so depending on the maximum allowed by the journal, the first five (for example) can be used if there is a need to reduce further.

You used abbreviations/acronyms at the abstract section.

**Response**

Thank you for bringing forth this observation. We were trying to restrict word count to maximum 300 which was the information we had. We have now revised the abstract by writing acronyms/abbreviations in full (Abstract, Page 2, lines 26-46; and Page 3, lines 50-51). The abstract is now 314-words long. We hope this is fine. We also just came across another PLOS One guide which says 300 words preferred by journal but maximum 500 words acceptable for abstracts.

Show clearly how you calculated sample size.

**Response**

Thank you. Details of sample size calculation has been added to the appropriate section under Methodology (Page 6, lines 124-141, and page 7, lines 142-152).

Why do you consider an attrition rate of 30%? How much is the recommendation?

**Response**

Thank you for this question. Generally, in clinical research, 10-20% is often used to adjust for attrition during sample size calculation. To the best of our knowledge there is no benchmark. Considering that this is a survey of health workers, we decided to use a higher value to adjust for non-response to improve our sample size for the following reasons: (i) Surveys are generally prone to higher rate of attrition compared to other forms of clinical research, and (ii) a previous study found that doctors are less likely to respond to surveys compared to patients (53% vs. 70%, p <0.05) irrespective of the mode of administering the survey and that average response rate for in-person surveys is 76%.^1^ (iii) When attrition is above 20% in a study, the risk of bias increases.^2^ To minimize these potential issues, we decided to adjust the sample size calculation for non-response of 30%.

Reference

1. Meyer VM, Benjamens S, Moumni ME, Lange JFM, Pol RA. Global Overview of Response Rates in Patient and Health Care Professional Surveys in Surgery: A Systematic Review. Annals of Surgery. 2022;275(1):e75-e81.
2. Dumville JC, Torgerson DJ, Hewitt CE. Reporting attrition in randomised controlled trials. Bmj. 2006;332(7547):969-71.

Put your operational definitions.

**Response**

Thank you. This has been added to the methods section (Page 10, lines 234-236, and page 11, lines 237-251).

More nurses/midwives (81.1%) compared to doctors (47.4%) reported lack of laboratory facilities for not screening for iron deficiency in anaemic pregnant women. Compared to doctors, a significantly higher percentage of nurses/midwives reported that they do not prescribe any treatment. Do you consider any knowledge and attitude issue in addition to referral practice?

**Response**

Thank you for this question. Yes, knowledge may be relevant in explaining this; generally, doctors have better exposure and thus knowledge regarding treatment of medical conditions. The curriculum for training doctors is more elaborate than that of the nurses. So, knowledge can partly explain why nurses are less likely to use the laboratory or prescribe treatment compared to doctor. We are not quite certain if attitude can play a role. Rather, health facility policies might contribute. Policies differ from facility to facility. Some health facilities may permit doctors to screen and treat certain conditions but not the nurses. Some of these comments not previously captured in the discussion have been added to the section (Page 23, lines 504-506).

Why do you concern in low usage of intravenous iron than oral iron?

**Response**

Thank you. For this study, there is no specific concern. This is one of the sub-studies for my (lead author) doctoral research. The main study was a randomized controlled trial which evaluated the effectiveness of intravenous iron (ferric carboxymaltose) and oral iron (ferrous sulphate) for the treatment of iron deficiency anaemia during pregnancy. So, we were interested in knowing more about the usage rate of intravenous iron in Nigeria.

**Reviewer #2:**

Thorough background research on the state of maternal health care workforce in Nigeria. Good job by the research team. More specific references for those values of the population of maternal healthcare professionals would be better.

**Response**

Thank you for this comment. Indeed, it would have been great if we were able to do so. Unfortunately, we tried to get data directly from the Medical and Dental Council of Nigeria for doctors, and Nurses and Midwifery Council of Nigeria for the nurses but did not succeed. So, we had to rely on available data. There is also no online source from which we could retrieve these data.
